# Assessment of brain tissue injury after moderate hypothermia in neonates with hypoxic–ischaemic encephalopathy: a nested substudy of a randomised controlled trial

**DOI:** 10.1016/S1474-4422(09)70295-9

**Published:** 2010-01

**Authors:** Mary Rutherford, Luca A Ramenghi, A David Edwards, Peter Brocklehurst, Henry Halliday, Malcolm Levene, Brenda Strohm, Marianne Thoresen, Andrew Whitelaw, Denis Azzopardi

**Affiliations:** a Perinatal Imaging, MRC Clinical Sciences Centre, Hammersmith Hospital, London, UK; b Neonatal Unit, Policlinico Mangiagalli, Fondazione Scientifica IRCCS, Milano, Italy; c Division of Clinical Sciences, Imperial College, and MRC Clinical Sciences Centre Hammersmith Hospital, London, UK; d National Perinatal Epidemiology Unit, University of Oxford, Old Road Campus, Oxford, UK; e Department of Child Health, Queens University, Belfast, Northern Ireland, UK; f Department of Paediatrics, University of Leeds, Leeds, UK; g Department of Child Health, St Michael's Hospital, Bristol, UK; h Department of Clinical Science, University of Bristol, Southmead Hospital, Bristol, UK

## Abstract

**Background:**

Moderate hypothermia in neonates with hypoxic–ischaemic encephalopathy might improve survival and neurological outcomes at up to 18 months of age, although complete neurological assessment at this age is difficult. To ascertain more precisely the effect of therapeutic hypothermia on neonatal cerebral injury, we assessed cerebral lesions on MRI scans of infants who participated in the Total Body Hypothermia for Neonatal Encephalopathy (TOBY) trial.

**Methods:**

In the TOBY trial hypoxic–ischaemic encephalopathy was graded clinically according to the changes seen on amplitude integrated EEG, and infants were randomly assigned to intensive care with or without cooling by central telephone randomisation. The relation between allocation to hypothermia or normothermia and cerebral lesions was assessed by logistic regression with perinatal factors as covariates, and adjusted odds ratios (ORs) were calculated. The TOBY trial is registered, number ISRCTN 89547571.

**Findings:**

325 infants were recruited in the TOBY trial between 2002 and 2006. Images were available for analysis from 131 infants. Therapeutic hypothermia was associated with a reduction in lesions in the basal ganglia or thalamus (OR 0·36, 95% CI 0·15–0·84; p=0·02), white matter (0·30, 0·12–0·77; p=0·01), and abnormal posterior limb of the internal capsule (0·38, 0·17–0·85; p=0·02). Compared with non-cooled infants, cooled infants had fewer scans that were predictive of later neuromotor abnormalities (0·41, 0·18–0·91; p=0·03) and were more likely to have normal scans (2·81, 1·13–6·93; p=0·03). The accuracy of prediction by MRI of death or disability to 18 months of age was 0·84 (0·74–0·94) in the cooled group and 0·81 (0·71–0·91) in the non-cooled group.

**Interpretation:**

Therapeutic hypothermia decreases brain tissue injury in infants with hypoxic–ischaemic encephalopathy. The predictive value of MRI for subsequent neurological impairment is not affected by therapeutic hypothermia.

**Funding:**

UK Medical Research Council; UK Department of Health.

## Introduction

Hypoxic–ischaemic encephalopathy after perinatal asphyxia is an important cause of mortality and morbidity in newborns that accounts for about 20% of occurrences of cerebral palsy in childhood.^1^, ^2^ Although antenatal factors have been implicated in the aetiology of hypoxic–ischaemic encephalopathy, little evidence of antenatal injury is seen on early MRI scans in neonates that present with hypoxic–ischaemic encephalopathy.^1^, ^3^, ^4^ Hypoxic–ischaemic encephalopathy can be graded on clinical grounds or according to the changes seen on amplitude integrated EEG.^5^, ^6^ The main neurodevelopmental sequela to moderate or severe hypoxic–ischaemic encephalopathy is severe neuromotor impairment, but cognitive and other impairments can also occur.^7^, ^8^, ^9^

Until recently, there were no specific treatments for hypoxic–ischaemic encephalopathy. The results of randomised controlled trials of 72 h of selective head or whole-body cooling started within 6 h of birth have suggested improved neurological outcomes up to 18 months of age, and treatment with hypothermia is increasingly used clinically.^10^, ^11^, ^12^, ^13^ Because neurological assessment at 18 months of age is difficult, other signs of the therapeutic efficacy of cooling would strengthen the evidence base for the treatment of hypoxic–ischaemic encephalopathy.

MRI is the optimum technique to detect perinatally acquired cerebral lesions, and the pattern and severity of the lesions provide a reliable guide to prognosis.^14^, ^15^, ^16^, ^17^, ^18^, ^19^, ^20^ In the presence of a sentinel event consistent with a severe acute hypoxic–ischaemic insult, lesions in the basal ganglia and thalami are often associated with abnormalities in specific cortical regions and in the adjacent subcortical white matter.^4^ Abnormal signal intensity in the posterior limb of the internal capsule coexists with lesions in the basal ganglia and thalami and is a powerful predictor of abnormal motor outcome.^14^ Lesions in the basal ganglia and thalami are often associated with abnormalities in specific cortical regions and the adjacent subcortical white matter after an event that is consistent with a severe acute hypoxic–ischaemic insult.^17^ Moderate and severe lesions in the basal ganglia and thalami and severe white matter lesions are associated with cerebral palsy.^4^, ^20^, ^21^

In the Total Body Hypothermia for Neonatal Encephalopathy (TOBY) trial, infants who were allocated to prolonged moderate hypothermia showed no significant difference in the primary outcome of death or disability at 18 months but had a reduced rate of cerebral palsy and improved mental and psychomotor outcomes compared with those allocated to standard care.^12^ We hypothesised that whole-body cooling would be associated with a reduction in cerebral lesions seen on MRI that are characteristic of hypoxic–ischaemic encephalopathy, including lesions that predict later neurodevelopmental impairments, and that cooling would not alter the accuracy of neonatal MRI for predicting neurological outcome at 18 months of age. To test this hypothesis, we reviewed the MRI scans obtained during the neonatal period in a nested substudy of the TOBY trial.

## Methods

### Patients

Infants born after 36 or more completed weeks' gestation who showed signs of moderate or severe encephalopathy, with or without seizures, were assessed with amplitude integrated EEG.^12^ Infants were randomly assigned, by central telephone randomisation within 6 h of birth, to a control group (rectal temperature kept to within 0·2°C of 37°C) or to whole-body cooling (rectal temperature kept at 33–34°C for 72 h). Mode of delivery, gestational age, sex, birthweight, Apgar score, occipitofrontal head circumference at birth, cooling status, and age at MRI scan were recorded.

The primary outcome of the TOBY trial was death or severe neurodisability at 18 months, which was defined as at least one from: mental development index (MDI) less than 70 (2 or more SD below the mean) on the Bayley infant scales (BSID II);^22^ a level of 3–5 on the gross motor function classification system (GMFCS), which ranges from 1 to 5, with 1 being the mildest impairment;^23^, ^24^ or bilateral cortical visual impairment with no useful vision.

The TOBY trial is registered, number ISRCTN 89547571, and was approved by the London Multicentre Research Ethics Committee and the local research ethics committee of each participating hospital. Written informed consent was obtained from one parent of each infant that participated in the trial.

### Procedures

Neonates were imaged with conventional T1-weighted and T2-weighted sequences at 1·5 T or 3 T. Images were included if they were acquired within the first 4 weeks after birth—the optimum period in which to image brain lesions that occurred around the time of birth.^16^ Two independent experts who were masked to treatment allocation reviewed the images for quality, normal anatomy, and acquired lesions. Images that were deemed to be of inadequate quality were not analysed further. The reviewers classified all images independently before the results were compared, and the inter-observer variability was estimated; cases of disagreement were resolved by consensus.

**Figure 1:**
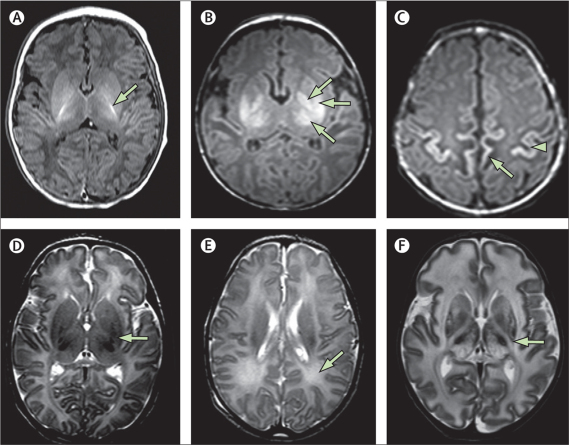
MRI appearances in neonatal hypoxic–ischaemic encephalopathy (A-C) T1-weighted images in the transverse plane. (A) Normal neonatal brain with linear high signal intensity representing myelin in the posterior limb of the internal capsule (arrow). (B) Moderate basal ganglia and thalamic lesions with abnormal increased signal intensity in the globus (top arrow), putamen (middle arrow), and thalamus (bottom arrow). There is no normal linear high signal intensity from the intervening posterior limb of the internal capsule. (C) Cortical lesions. There is abnormal increased signal intensity in the cortex around the central sulcus (arrow head) and along the interhemispheric fissure. Abnormal low signal intensity is seen in the adjacent subcortical white matter (arrow). (D–F) T2-weighted images in the transverse plane. (D) Mild lesions in the basal ganglia. There are small bilateral foci of abnormal low signal intensity in the lentiform nuclei (arrow). (E) Mild white matter lesions. There is diffuse and slightly increased signal intensity in the periventricular white matter (arrow). (F) Severe basal ganglia and thalamic and white matter lesions. There is mixed abnormal signal intensity throughout the basal ganglia and thalami. The intervening posterior limb of the internal capsule shows abnormal high signal intensity with no evidence of low signal intensity from myelin (arrow). The hemispheric white matter has diffuse abnormal high signal intensity throughout.

A diagnosis of venous sinus thrombosis was made if abnormal signal intensity was seen in a major venous sinus and was associated with lesions that were consistent with haemorrhagic venous infarction in the draining territory of the sinus. Subdural haemorrhage was classified as mild, moderate, or severe. The pattern of injury was classified according to the abnormalities seen, as previously described (webappendix).^17^ Moderate or severe lesions in the basal ganglia and thalamus, an abnormal posterior limb of the internal capsule, or severe white matter lesions were deemed to be predictive of abnormal neurodevelopmental outcome (figure 1).

### Statistical analysis

Data were analysed with SPSS version 16 (SPSS, IL, USA). Clinical characteristics between groups were compared by χ^2^ for categorical data and *t* test or Mann-Whitney test, as appropriate, for continuous data. The relations among sex, gestational age, postnatal age at scan, Apgar score, severity of hypoxic–ischaemic encephalopathy seen on amplitude integrated EEG, treatment allocation, and cerebral lesions seen on MRI were assessed by univariate analysis. Logistic regression and calculation of adjusted odds ratios (adjusted OR, 95% CI) were done to assess the effect of cooling on cerebral lesions seen on MRI, controlling for the perinatal factors that were significant on univariate analysis. Two-sided p values less than 0·05 were regarded as significant.

The ability of MRI to predict an abnormal outcome, defined as death or severe disability at 18 months of age, was assessed for both cooled and non-cooled infants by calculation of the predictive accuracy, sensitivity, specificity, and positive and negative predictive values.

### Role of funding source

The study sponsors had no role in the study design, data collection, data analysis, data interpretation, or writing of the report. The corresponding author had full access to all the data in the study and had final responsibility for the decision to submit the paper for publication.

## Results

**Figure 2:**
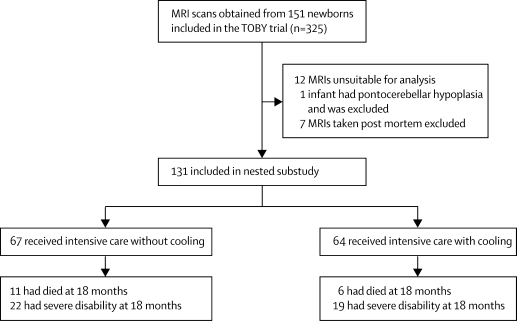
Study profile

325 infants were recruited to the TOBY trial between 2002 and 2006. MRI scans were obtained from 151 infants from the 22 hospitals with facilities for neonatal MRI. Figure 2 shows the study profile. 12 (seven cooled, five non-cooled) of the 151 sets of images were unsuitable for analysis. One infant who had an overt pontocerebellar hypoplasia seen on MRI was excluded from further analysis. MRIs from three cooled and four non-cooled infants were taken post mortem, and these images were not included. Therefore, images were available for analysis for 131 infants: 64 in the group allocated to cooling and 67 infants in the non-cooling group. There was no difference in gestational age or birthweight between the two groups. The cooled infants had similar 10-min Apgar scores and there was no difference in the severity of amplitude integrated EEG between the two groups (p=0·32; table 1). The clinical characteristics of these 131 infants were not different from the rest of the infants in the TOBY trial, although there were more severely abnormal amplitude integrated EEG findings in infants that were not included in the substudy (webappendix).

**Table 1:** Clinical characteristics of the infants

		**Cooled (n=64)**	**Non-cooled (n=67)**	**p**
Gestational age (weeks)		40 (39–41)	40 (39–41)	0·73
Birthweight (g)		3450 (2995–3863)	3290 (3075–3736)	0·30
Occipitofrontal circumference at birth (cm)		35·3 (34·1–36·1)	35 (34·1–35·6)	0·61
Boys:girls		39:25	35:32	0·32
Apgar score at 10 min		4 (3–5)	4 (3–5)	0·36
Complications during delivery		48 (75%)	51 (76%)	0·76
Amplitude integrated EEG				
	Moderately abnormal	27 (42%)	34 (51%)	0·32*
	Severely abnormal	35 (55%)	31 (46%)	
Age at postnatal scan (days)		8 (6–11)	8 (5–10)	0·27

Data are median (IQR) or n (%). Amplitude integrated EEG was not available for two infants in each group.

* χ^2^ test for the proportion of moderately abnormal to severely abnormal amplitude integrated EEG in cooled (27:35) and non-cooled (34:31) groups.

The median age at postnatal scan was 8 days (range 2–30) in both groups and all infants were older than 37 weeks gestational age at the time of scan. Observer agreement for classification of MRI scans was greater than 99%. None of the patterns of injury were deemed to be unusual for patients with hypoxic–ischaemic encephalopathy.

On univariate analysis, treatment allocation was significantly associated with abnormal MRI in the basal ganglia and thalami (p=0·01), white matter (p=0·01), and the posterior limb of the internal capsule (p=0·03); there was an improvement of trend across categories of severity of MRI abnormalities in the basal ganglia and thalami (p=0·02) and white matter (p=0·02) in the cooled group compared with the non-cooled group. Postnatal age at scan was significantly associated with abnormalities in the basal ganglia and thalami when examined as a continuous variable (p=0·02) or categorised into less than 8 days or 8 or more days (p=0·01), but did not correlate with abnormalities in the white matter or cortex. The amplitude integrated EEG grade correlated with abnormalities in the basal ganglia and thalami (p=0·001), white matter (p=0·003), and posterior limb of the internal capsule (p<0·0001). Postnatal age at scan and grade of amplitude integrated EEG were included as covariates in all the binomial logistic regression analyses of treatment allocation and MRI findings (table 2).

**Table 2:** Grades of cerebral lesions seen on MRI in cooled and non-cooled infants

		**Cooled (n=64)**	**Non-cooled (n=67)**	Adjusted*		Unadjusted*	
				OR (95% CI)	p	OR (95% CI)	p
Basal ganglia and thalami							
	0	26	14	0·36 (0·15–0·84)	0·02	0·39 (0·18–0·84)	0·02
	1	11	14				
	2	11	14				
	3	16	25				
Posterior limb of internal capsule							
	Normal	34	23	0·38 (0·17–0·85)	0·02	0·46 (0·23–0·93)	0·03
	Equivocal	2	5				
	Abnormal	28	39				
White matter							
	Normal	23	11	0·30 (0·12–0·77)	0·01	0·35 (0·15–0·80)	0·01
	1	19	26				
	2	15	21				
	3	7	9				
Cortex†							
	0	34	24	0·62 (0·27–1·41)	0·25	0·65 (0·29–1·42)	0·28
	1	16	22				
	2	10	16				
	3	4	4				
Intracranial haemorrhage		25	22	Not done		1·31 (0·64–2·68)	0·11

Data are number or OR (95% CI).

* Odds ratio for presence or absence of MRI abnormalities in cooled and non-cooled infants, with and without adjustment for severity of amplitude integrated EEG and postnatal age. OR=odds ratio.

† Cortex could not be assessed in one infant in the non-cooled group.

Lesions in the basal ganglia and thalami were detected in 38 of 64 (59%) cooled infants and 53 of 67 (79%) non-cooled infants (adjusted OR 0·36, 95% CI 0·15–0·84; p=0·02, table 2). The posterior limb of the internal capsule was normal in 34 of 64 (53%) cooled infants and 23 of 67 (34%) non-cooled infants, equivocal in two of 64 (3%) cooled infants and five of 67 (7%) non-cooled infants, and abnormal in 28 of 64 (44%) cooled infants and 39 of 67 (58%) non-cooled infants (0·38, 0·17–0·85; p=0·02). White matter abnormalities were seen in 41 of 64 (64%) cooled infants and 56 of 67 (84%) non-cooled infants (0·30, 0·12–0·77; p=0·01). Cortical abnormalities were seen in 30 of 64 (47%) cooled infants and 42 of 66 (64%) non-cooled infants (0·62, 0·27–1·41; p=0·25).

Lesions that predicted abnormal neurodevelopmental outcome (defined as at least one of: moderate or severe lesions in the basal ganglia and thalami [grade 2 or 3], an abnormal posterior limb of the internal capsule, or severe white matter abnormalities [grade 3]) were seen in 29 of 64 (45%) cooled infants and 42 of 67 (63%) non-cooled infants (adjusted OR 0·42, 95% CI 0·20–0·92; p=0·03). 22 of 64 (34%) cooled infants and 11 of 67 (16%) non-cooled infants had normal scans (2·81, 1·13–6·93; p=0·03).

Logistic regression analysis showed that, in addition to treatment allocation, grade of amplitude integrated EEG was associated with abnormalities in the basal ganglia and thalami, white matter, and posterior limb of the internal capsule (all p<0·0001); major abnormalities that predicted abnormal outcome (p<0·0001); and normal scans (p=0·001). 37 of 59 (63%) infants scanned at less than 8 days of age had major abnormalities on MRI that were predictive of abnormal outcome compared with 34 of 72 (47%) infants scanned later (p=0·08; table 3). After adjustment for grade of amplitude integrated EEG and treatment allocation in infants scanned at less than 8 days of age compared with those scanned later, the OR for the detection of major abnormalities seen on MRI was 2·35 (1·07–5·14; p=0·03).

**Table 3:** Postnatal age at scan, MRI abnormalities, and outcome up to 18 months of age in cooled and non-cooled infants

		Cooled*(n=63)	**Non-cooled (n=67)**	**Total**
**<8 days**				
MRI abnormalities absent				
	Not disabled	10	11	21
	Died or disabled	0	1	1
	Total	10	12	22
MRI abnormalities present				
	Not disabled	1	7	8
	Died or disabled	16	13	29
	Total	17	20	37
**≥8 days**				
MRI abnormalities absent				
	Not disabled	21	12	33
	Died or disabled	3	1	4
	Total	24	13	37
MRI abnormalities present				
	Not disabled	6	4	10
	Died or disabled	6	18	24
	Total	12	22	34

Data are number of infants. Major MRI abnormalities were defined as moderate or severe basal ganglia or thalamic lesions, severe white matter lesions, or abnormal posterior limb of the internal capsule. Severe disability was defined as at least one of the following: mental development index (MDI) score less than 70 (≥2 SD below the mean) on the Bayley Infant Scales (BSID II); score of 3–5 on the gross motor function classification system (GMFCS), which ranges from 1 to 5, with 1 being the mildest impairment; or bilateral cortical visual impairment with no useful vision.

* Outcome at 18 months was not available for one infant in the group allocated to cooling.

Three infants (two cooled and one non-cooled) had evidence of sinus thrombosis and 47 (36%) had evidence of haemorrhage: 25 from the cooled group and 22 from the non-cooled group. The haemorrhage was subdural in 39 infants, moderate in ten infants, and mild in 29 infants.

Assessment of the primary outcome was available for all but one infant. Of 130 infants, six cooled and 11 non-cooled infants died (p=0·23), whereas 19 cooled and 22 non-cooled infants had severe disability at 18 months of age (p=0·70; table 3). In the main TOBY trial,^12^ the rates of death were 42 of 163 (0·26) in the cooled group and 44 of 162 (0·27) in the non-cooled group. The rates for severe disability in survivors were 32 of 120 (0·27) in the cooled group and 42 of 117 (0·36) in the non-cooled group.

In this subset, all 17 infants who died and 36 of 41 (88%) infants with severe disability had moderate or severe lesions in the basal ganglia and thalami and an abnormal posterior limb of the internal capsule. Four of the remaining five infants who were classified as having severe disability had mild lesions in the basal ganglia and thalami and mild or normal white matter; the other infant had normal basal ganglia and thalamus but moderate lesions in the white matter. These five infants had an MDI less than 70 but a psychomotor developmental index (PDI) greater than 70. Four of the five infants had a GMFCS of 1 (the mildest level of impairment).

11 infants with moderate lesions and two infants with severe lesions in the basal ganglia and thalami did not have severe disability at 18 months. An abnormal posterior limb of the internal capsule was seen in 11 of 13 infants. These 13 infants had a PDI and MDI greater than 70, but five had a GMFCS of 1. All 34 infants (22 cooled, 12 non-cooled) with normal basal ganglia and thalami and normal or mild lesions in the white matter had a normal neuromotor outcome at 18 months.

The predictive accuracy of moderate or severe lesions in the basal ganglia and thalami, severe white matter lesions, or an abnormal posterior limb of the internal capsule for death or severe disability at 18 months of age was 0·84 (95% CI 0·74–0·94 [53 of 63]) in the cooled group and 0·81 (0·71–0·91 [54 of 67]) in the non-cooled group (table 4).

**Table 4:** Predictive ability of major MRI abnormalities during first 4 weeks after birth for death or severe disability at 18 months

	**Cooled**	**Non-cooled**
Sensitivity	0·88 (0·79–0·97)	0·94 (0·88–1·0)
Specificity	0·82 (0·72–0·92)	0·68 (0·56–0·80)
Positive predictive value	0·76 (0·65–0·87)	0·74 (0·63–0·85)
Negative predictive value	0·91 (0·83–0·99)	0·92 (0·85–0·99)

Data are proportions (95% CI). Major MRI abnormalities were defined as moderate or severe basal ganglia or thalamic lesions, severe white matter lesions, or an abnormal posterior limb of the internal capsule.

## Discussion

We report brain MRI findings from a subgroup of infants enrolled in a randomised controlled trial of therapeutic hypothermia in neonates with hypoxic–ischaemic encephalopathy. Therapeutic hypothermia was associated with less grey and white matter abnormalities, and more cooled than non-cooled infants had normal MRI scans. The accuracy of MRI done during the neonatal period for the prediction of neurological outcomes up to 18 months of age was unaltered by therapeutic hypothermia. In this large cohort of infants who had an MRI after hypoxic–ischaemic encephalopathy, we found no unusual patterns of lesions and no increase in haemorrhagic or thrombotic lesions associated with therapeutic hypothermia.

These results are likely to be reliable because the clinical characteristics of the study infants were not different from the other infants in the TOBY trial; almost all infants (87% [131 of 151]) in TOBY who had MRI were included in the analysis; perinatal factors were similarly distributed among those allocated to therapeutic hypothermia or normothermia; and perinatal factors that were associated with MRI were included in a logistic analysis to assess any independent effect of therapeutic hypothermia. The ORs for predicting MRI abnormalities by treatment allocation were not significantly different from those calculated by univariate analysis (table 2).

As expected, the mortality rate in the study infants was less than that in the TOBY trial because many deaths occurred before MRI could be done, which is also likely to be the case in clinical practice. These differences do not alter the study results because the mortality rate did not differ significantly between the cooled and non-cooled infants and was similar to the results of the main TOBY trial; furthermore, mortality was included in the assessment of the prognostic accuracy of MRI.

Systematic reviews of randomised trials have shown improved survival and neurological outcomes after therapeutic hypothermia in newborns with hypoxic–ischaemic encephalopathy.^25^ However, only three trials have reported outcomes to 18 months of age and the study design and outcomes differed among the trials: the selective head cooling with mild systemic hypothermia after neonatal encephalopathy trial (the Coolcap trial)^11^ used selective head cooling with mild whole-body cooling, whereas the whole-body hypothermia for neonates with hypoxic–ischaemic encephalopathy trial (the NICHD trial)^12^ and the TOBY trial used whole-body cooling. Only the NICHD trial showed a significant reduction in the primary outcome of the combined rates of death and disability. The finding of a reduction in cerebral lesions on MRI that are associated with subsequent neurodevelopmental abnormalities and a higher rate of normal scans in cooled compared with non-cooled infants strengthens the evidence for the neuroprotective efficacy of prolonged moderate hypothermia in newborns with hypoxic–ischaemic encephalopathy.

Two observational studies have assessed MRIs of infants treated with hypothermia after hypoxic–ischaemic encephalopathy, although neither study controlled for perinatal factors that might affect findings on MRI.^26^, ^27^ In one non-randomised study of hypoxic–ischaemic encephalopathy in 34 infants treated with either head cooling or whole-body cooling and 52 non-cooled infants, there was a reduction in the incidence of moderate and severe lesions in the basal ganglia and thalami with cooling but this was only significant in infants with an early moderately abnormal amplitude integrated EEG.^27^ Fewer cortical abnormalities were seen in the head-cooling group but not in the group treated with whole-body cooling. In another study of 26 infants, whole-body cooling was associated with fewer cortical abnormalities than in the controls, suggesting a preferential effect of cooling on the cortex.^26^ However, in our study cooling was associated with significant reductions in abnormalities in the basal ganglia and thalami and the white matter, but the reduction in cortical grey matter abnormalities in the cooled compared with the non-cooled group was not statistically significant (table 2).

Therapeutic hypothermia might alter the prognostic evaluation of infants with hypoxic–ischaemic encephalopathy.^28^ Secondary analysis of the Coolcap study showed that infants treated with cooling had a more favourable prognosis than was suggested by the clinical grade of encephalopathy compared with infants treated with standard care. Therefore, the prognostic value of neuroimaging might be altered after therapeutic hypothermia. A comparison of prognostic accuracy requires that the tests are compared in the same population and is therefore not applicable in our study. However, we found that the prediction of the combined rates of death and severe disability to 18 months of age by MRI was good in cooled and non-cooled infants and was similar to that previously reported for infants who received standard care without cooling.^14^, ^20^

MRI findings after hypoxic–ischaemic encephalopathy might be affected by the postnatal age at scan. The characteristic abnormalities in the grey and white matter might not be apparent on conventional T1-weighted or T2-weighted imaging until some days after birth, but might be seen earlier with diffusion-weighted imaging.^29^ We found that infants who were younger than 8 days when scanned were more likely to have major MRI abnormalities. Possible explanations are that MRI might be done earlier in infants with more severe encephalopathy to direct clinical care; alternatively, abnormalities detected during the first few days after birth might subsequently resolve spontaneously. Because we were unable to assess which factors, such as illness severity and access to scanning, affected the timing of MRI scans, it is not possible to ascertain from these data the optimum period during which conventional MRI should be done after hypoxic–ischaemic encephalopathy. Because no additional resources for MRI scanning were provided to the participating hospitals, our finding that MRI at a median of 8 days accurately predicted outcome at 18 months of age in cooled and non-cooled infants is likely to be generally applicable.

These data show that MRI in the neonatal period is qualified as a biomarker of disease and treatment response and might be of use in other neuroprotective studies.

## Web Extra Material

Supplementary webappendix
