# Supplementary material for: Assessment of brain tissue injury after moderate hypothermia in neonates with hypoxic–ischaemic encephalopathy: a nested substudy of a randomised controlled trial
Source: Lancet Neurol. 2010 Jan;9(1):39–45. doi: 10.1016/S1474-4422(09)70295-9 (PMC2795146; doi:10.1016/S1474-4422(09)70295-9)
Supplement: Supplementary webappendix [file mmc1.pdf]

THE LANCET Neurology

Supplementary webappendix

This webappendix formed part of the original submission and has been peer reviewed. We post it as supplied by the authors.

Supplement to: Rutherford M, Ramenghi LA, Edwards AD, et al. Assessment of brain tissue injury after moderate hypothermia in neonates with hypoxic–ischaemic encephalopathy: a nested substudy of a randomised controlled trial. *Lancet Neurol* 2009; published online November 6. DOI:10.1016/S1474-4422(09)70295-9.

Web appendix

Image visual analysis

- Normal anatomy. The presence of any congenital abnormalities was documented. Such images were assessed for the presence of acquired injury but were excluded from further visual analysis—eg, agenesis of the corpus callosum, developmental cerebellar anomalies, abnormal configuration of the cortex
- The presence of abnormalities thought to have resulted from an injury occurring prior to labour and delivery—eg, established cysts seen during the first week, marked ventricular dilatation with white matter atrophy, abnormal signal intensity thought to be older than perinatal in origin
- Abnormal signal intensity within the following areas: posterior limb of the internal capsule, thalami, and the basal ganglia (putamen, globus pallidus, caudate nucleus)
- Abnormal signal intensity within the white matter and cortex
- Appearance of the medial temporal lobe, specifically the hippocampus
- Appearance of the corpus callosum—ie, abnormal signal intensity or atrophy
- Abnormal appearance to the extracerebral space—eg, presence of extracerebral haemorrhage or marked widening
- Appearance of the ventricles: 1=normal, 2=mild dilation obvious asymmetry, 3=marked dilatation
- Appearance of the brainstem including abnormal signal intensity and/or asymmetry
- Appearance of the cerebellum—eg, abnormal signal intensity, focal lesions, abnormal size
- Presence and site of haemorrhage
- Presence and site of sinus or venous thrombosis

Pattern of injury score

Posterior limb of the internal capsule (PLIC) score: 0=normal, 1=equivocal (reduced or asymmetrical signal intensity), 2=loss (reversed or abnormal signal intensity bilaterally on T1 and/or T2 weighted sequences).

Basal ganglia and thalamic (BGT) score: 0=normal, 1=mild (focal abnormal signal intensity), 2=moderate (multifocal abnormal signal intensity), 3=severe (widespread abnormal signal intensity).

White matter (WM) score: 0=normal, 1=mild (exaggerated long T1 and long T2 in periventricular white matter only), 2=moderate (long T1 and long T2 extending out to subcortical white matter and /or focal punctate lesions or focal area of infarction), 3=severe widespread abnormalities including overt infarction, haemorrhage, and long T1 and long T2. Infarction was classified as areas of excessive long T1 and long T2 with either loss of grey-white matter differentiation (usually within first week exaggerated grey-white matter differentiation after the first week). Optic radiation was scored as abnormal if it demonstrated increased signal intensity on T1 weighted images.

Cortical involvement was scored as the presence of abnormal signal intensity, usually decreased T1 or cortical highlighting. 0=normal, 1=mild (1–2 sites involved), 2=moderate (3 sites involved), 3=severe (more than 3 sites involved). The sites documented included specifically the central sulcus, interhemipsheric fissure, and the insula.

Each infant was then given a score for PLIC, BGT, WM, and cortex.

Patient characteristics of infants without MRI data

Characteristic	Cooled (n=99)	Not cooled (n=95)
Male sex	61 (62%)	54 (57%)
Gestational age (weeks)	40 (37.6–42.4)	40.1 (37.7–42.5)
Birthweight (g)	3450 (2526–4374)	3350 (2591–4109)
Head circumference (cm)	35.0 (33.0–37.0)	35.0 (33.2–36.8)
Delivery complications	67 (67.7%)	67 (70.5%)
Apgar score at 10 min	4 (2–5)	4 (2–5)
Amplitude integrated EEG		
Moderately abnormal	38 (38.4%)	34 (35.8%)
Severely abnormal	61 (61.6%)	61 (64.2%)

Data are n (%) or median (IQR).

p values for comparisons between infants with MRI data and infants without MRI data

Characteristic	Cooled	Not cooled
Male sex	0.93	0.56
Gestational age (weeks)	0.71	0.71
Birthweight (g)	0.98	0.72
Head circumference (cm)	0.58	0.92
Delivery complications	0.32	0.34
Apgar score at 10 min	0.93	0.93
Amplitude integrated EEG		
Moderately abnormal	0.52	0.05
Severely abnormal		
